# DNA Damage Repair Deficiency in Pancreatic Ductal Adenocarcinoma: Preclinical Models and Clinical Perspectives

**DOI:** 10.3389/fcell.2021.749490

**Published:** 2021-10-12

**Authors:** Jojanneke Stoof, Emily Harrold, Sarah Mariottino, Maeve A. Lowery, Naomi Walsh

**Affiliations:** ^1^Trinity St. James Cancer Institute, Trinity College Dublin, Dublin, Ireland; ^2^Trinity College Dublin, Dublin, Ireland; ^3^Mater Private Hospital, Dublin, Ireland; ^4^National Institute of Cellular Biotechnology, School of Biotechnology, Dublin City University, Dublin, Ireland

**Keywords:** DNA damage response (DDR), pancreatic duct adenocarcinoma (PDAC), preclinical model, cell line, organoid, genetically engineered mouse model (GEMM), xenograft, targeted therapy

## Abstract

Pancreatic ductal adenocarcinoma (PDAC) is one of the most lethal cancers worldwide, and survival rates have barely improved in decades. In the era of precision medicine, treatment strategies tailored to disease mutations have revolutionized cancer therapy. Next generation sequencing has found that up to a third of all PDAC tumors contain deleterious mutations in DNA damage repair (DDR) genes, highlighting the importance of these genes in PDAC. The mechanisms by which DDR gene mutations promote tumorigenesis, therapeutic response, and subsequent resistance are still not fully understood. Therefore, an opportunity exists to elucidate these processes and to uncover relevant therapeutic drug combinations and strategies to target DDR deficiency in PDAC. However, a constraint to preclinical research is due to limitations in appropriate laboratory experimental models. Models that effectively recapitulate their original cancer tend to provide high levels of predictivity and effective translation of preclinical findings to the clinic. In this review, we outline the occurrence and role of DDR deficiency in PDAC and provide an overview of clinical trials that target these pathways and the preclinical models such as 2D cell lines, 3D organoids and mouse models [genetically engineered mouse model (GEMM), and patient-derived xenograft (PDX)] used in PDAC DDR deficiency research.

## Introduction

Pancreatic cancer is one of the most lethal cancers worldwide, accounting for 2.6% of all new cancer cases but causing 4.8% of all cancer deaths (Ferlay et al., 2019). Despite recent advances in personalized and targeted therapy, little progress has been made to improve overall survival (OS) and the 5-year survival rate is estimated at 9% (Siegel et al., 2020).

Currently, curative treatment is limited to low-stage, resectable disease but over 80% of patients present with advanced or metastatic disease (Bilimoria et al., 2007; Stathis and Moore, 2010; Ilic and Ilic, 2016). Current standard of care treatment for advanced pancreatic ductal adenocarcinoma (PDAC) consists of either combination treatment of nab-paclitaxel with gemcitabine or 5-fluorouracil, leucovorin, irinotecan, oxaliplatin (FOLFIRINOX) (Mohammed et al., 2014; Mohammad, 2018; Adel, 2019). In 2013, the MPACT trial showed that nab-paclitaxel with gemcitabine improved OS by 1.8 months compared to gemcitabine alone (8.5 vs. 6.7 months, *p* < 0.001) (Von Hoff et al., 2013). The PRODIGE trial found that FOLFIRINOX improved OS by 4.3 months compared to gemcitabine alone (11.1 vs. 6.8 months, *p* < 0.001) (Conroy et al., 2011). However, FOLFIRINOX is associated with higher toxicity profiles and is therefore generally reserved for patients with a good performance status and given as a modified regimen. Systemic treatment options for PDAC include gemcitabine, gemcitabine with erlotinib, FOLFIRINOX, gemcitabine with nab-paclitaxel, nano-liposomal irinotecan with 5-FU, pembrolizumab (patients with microsatellite instability), larotrectinib/entrectinib (patients with NTRK-fusion), and olaparib (patients with g*BRCA* mutation).

Genomic analyses have revealed a complex mutational landscape that is predominated by mutations in *TP53*, *KRAS*, *SMAD4*, and *CDKN2A* (Bailey et al., 2016; Aguirre et al., 2018). Despite extensive research, targeted therapies for these mutations have not reached clinical practice (Qian et al., 2020). In addition, PDAC is characterized by genome instability (Alexandrov et al., 2013). Genome instability has been described as one of the enabling hallmarks of cancer by Hanahan and Weinberg (2011) and can be attributed to multiple sources, including increased sensitivity to mutagenic agents, defects in the genomic maintenance machinery, loss of telomeric DNA, and aberrant surveillance mechanisms. While these aberrations can partly be contributed to these four commonly mutated genes, additional pathway deficiencies are also involved. The DNA damage response (DDR) pathway plays a central role in genome maintenance and repair. In contrast to *TP53* and *KRAS*, DDR deficiency is targetable, with multiple drugs already available in the clinic for non-PDAC cancer types, such as breast and prostate cancer (Wengner et al., 2020).

This review briefly covers the role and definition of DDR deficiency in PDAC and provides an overview of clinical trials that investigate DDR targeting drugs. The main focus is on how cell lines, organoids, and mouse models are used to study DDR deficient pathways in PDAC.

## Main

### DNA Damage Repair Pathways in Pancreatic Ductal Adenocarcinoma

**FIGURE 1:**
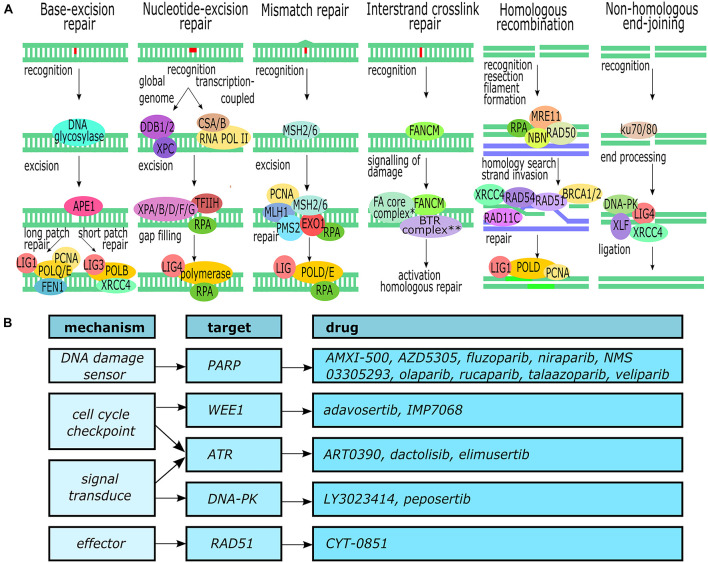
DNA damage repair. **(A)** Overview of the major DNA repair pathways (Deans and West, 2011; Yang et al., 2016; Ranjha et al., 2018; Gupta and Heinen, 2019; Lee and Kang, 2019). ^∗^The Fanconi anemia core complex consists of *FANCA*, *FANCB*, *FANCC*, *FANCD*, *FANCE*, *FANCF*, *FANCG*, and *FAAP100*. ^∗∗^The BTR complex consists of *BLM*, *TOPOIII*, *RMI1*, and *RMI2*. **(B)** Molecular targets within the DDR pathways and available inhibitors. This figure was created in Inkscape.

To combat the development of mutations and the effects these may have on the cell a complex network of DNA damage repair (DDR) pathways exists (Giglia-Mari et al., 2011). At the core of this network are the pathways for base excision repair (BER), nucleotide excision repair (NER), mismatch repair (MMR), interstrand crosslink repair (ICL repair), and double strand break repair [both homologous recombination (HR) and non-homologous end-joining (NHEJ)] (Figure 1). Each pathway can roughly be divided into three phases or steps: recognition of the damage, excision or processing of the damaged strand(s), and the actual repair.

Base excision repair removes non-bulky single-base lesions such as oxidation or deamination damage (Lee and Kang, 2019). The damaged base is recognized and removed by one of multiple specific DNA glycosylases (such as UNG, SMUG1, or NEIL1), depending on the type of lesion. Next, the newly created abasic site is excised and processed by APE1 to generate a 3′-hydroxyl site. This 3′-hydroxyl is then used by DNA polymerase to fill the gap using the opposing strand as template.

Nucleotide excision repair is the main pathway for the removal of bulky lesions but can also remove intrastrand crosslinks and cyclobutene pyrimidine dimers that are produced by UV radiation (Schärer, 2013; Lee and Kang, 2019). While two subpathways can be distinguished – global genome NER (GG-NER) for the whole genome and transcription-coupled NER (TC-NER) for the transcribing strand of active genes, the general repair process is similar to BER. GG-NER recognizes distortions of the DNA helix through DDB1, DDB2, and XPC, whereas TC-NER CSA and CSB recognize blockage of the RNA polymerase. TFIIH opens up the DNA to enable XPD to verify the lesion upon which several other XP endonucleases and RPA are recruited to excise the lesion. Finally, the resulting 22–32 nt long gap is filled and ligated to the original DNA strand by DNA polymerases and ligases.

The MMR pathway removes single nucleotide mismatches and small insertions or deletions created by DNA polymerase during DNA synthesis (Gupta and Heinen, 2019). The lesions are recognized by the heterodimer MSH2/MSH6. The dimer recruits another heterodimer, MLH1–PMS2, and together they recruit several other proteins including Exo1 to excise the damage. Finally, polymerase eta or delta fills the newly created gap.

Interstrand crosslinks (ICLs) are caused by bifunctional alkylating agents that form covalent bonds between the two DNA strands (Deans and West, 2011; Hashimoto et al., 2016). In quiescent cells the lesion is recognized and repaired by the NER pathway, but during the S phase several steps take place to activate the HR pathway. When a DNA replication fork encounters an ICL the fork stalls and, through a complex containing FANCM, the lesion is recognized and the Fanconi anemia complex and BTR complex are recruited. These complex create a double-strand break (DSB) which is subsequently recognized and repaired by the HR pathway.

Double-strand breaks are repaired through two main pathways: HR and NHEJ (Giglia-Mari et al., 2011; Yang et al., 2016; Ranjha et al., 2018). HR can take place during the S- and G2-phase of the cell cycle when it can use the homologous sequence of a sister chromatid to accurately repair the break. The DSB is recognized by the MRN complex (consisting of MRE11, RAD50, and NBN), the ends of the break are resected, and RPA binds to and forms a filament between the newly resected single-stranded DNA section. Next, BRCA2 recruits RAD51 to replace the RPA-filament and, assisted by several other proteins, homology search and strand invasion of the sister chromatid takes place. Using the sister chromatid as template, polymerase delta synthesizes the missing nucleotides of the broken strand and the ends are ligated. NHEJ, in contrast, can take place during every phase of the cell cycle and is quicker than HR but is also error-prone and commonly results in small deletions. The break is recognized by the ku70/80 heterodimer which subsequently recruits DNA-PKcs, XLF, XRCC4, and Lig4 to process and ligate the broken ends of the DNA strands. In recent years, important progress has been made in deciphering the molecular underpinnings of PDAC due to the unparalleled power of next generation sequencing (NGS) technologies. Constitutive mutations of PDAC have been described as selective DDR pathways in PDAC, however, the main problem encountered is the heterogeneity of somatic alterations among patients outside of the four most frequently mutated genes (KRAS, CDKN2A, TP53, and SMAD4) which poses a challenge to the identification of potential predictive and prognostic biomarkers.

#### Germline Mutations

Approximately 10% of all PDAC cases are considered familial; defined as a family with at least two first-degree relatives with PDAC (Turati et al., 2013). While several germline pathogenic alterations that increase an individual’s lifetime risk of PDAC (e.g., hereditary pancreatitis and Lynch syndrome) have been characterized, the causative germline mutation of most familial cases remains unclear (Klein, 2012). The most commonly mutated genes in familial pancreatic cancer are *BRCA2*, *CDKN2A*, *BRCA1*, and *PALB2* (Perkhofer et al., 2020). Pathogenic germline alterations have also been identified in patients who do not meet criteria for familial PDAC, and may involve genes beyond those previously associated with hereditary pancreatic cancer. These pathogenic germline alterations are therapeutically considered actionable in 5–10% of patients, and clinical guidelines now support routinely offering germline genetic testing with a broad panel of known hereditary cancer predisposition genes to all PDAC patients.

#### Somatic Mutations

The presence of DDR gene mutations has been reported in 17–43% of all sporadic PDAC patients (Waddell et al., 2015; Aguirre et al., 2018). However, these papers focused on a limited selection of well-characterized DDR genes and potentially actionable DDR mutations may be more prevalent. We queried the GENIE cohort (The AACR Project GENIE Consortium, 2017) containing 3706 PDAC patients with somatic mutation profiling for the presence of mutations in any of the genes of the six major DDR pathways (BER, NER, HR, NHEJ, ICL repair, and MMR) (Deans and West, 2011; Yang et al., 2016; Ranjha et al., 2018; Gupta and Heinen, 2019; Lee and Kang, 2019). A comprehensive list of 352 genes was collated based on the gene lists of the respective pathways in the Gene Ontology database. Mutations were reported in 117 (33%) of the genes, with 46 (13%) and 14 (4.0%) genes being mutated in more than 1 and 2% of the patients, respectively. The most commonly mutated genes were *TP53* (68.9%), *BRCA2* (4.4%), *ATM* (4%), and *PRKDC* (3.9%). An overview of these genes and associated pathways can be found in Table 1, Figure 1, and Supplementary Table 1.

**TABLE 1:** Prevalence of the top 25 most frequently found somatic DDR gene mutations and associated pathways in a cohort of 3706 PDAC patients.

Gene	Mutation frequency (%)	Affected pathways (respective GO term)					
		HR (GO:724)	NHEJ (GO:6303)	BER (GO:6284)	NER (GO:6289)	ICL repair (GO:36297)	MMR (GO:6298)
*TP53*	68.90				x		
*BRCA2*	4.40	x			x		
*ATM*	4.00		x				
*PRKDC*	3.90		x				
*MCM4*	3.50	x					
*NIPBL*	3.20	x					
*POLQ*	3.20	x	x	x			
*RIF1*	3.10	x	x				
*WRN*	2.40	x		x			
*FAAP100*	2.40					x	
*FANCD2*	2.40					x	
*ERCC6*	2.20	x	x	x	x		
*EP300*	2.10				x		
*RECQL4*	2.00	x					
*HELQ*	1.90	x					
*CUL4A*	1.80				x		
*ARID2*	1.80	x					
*FANCM*	1.80	x				x	
*FANCA*	1.80					x	
*PAXIP1*	1.70		x				
*FAN1*	1.60	x			x	x	
*BRCA1*	1.60	x	x				
*MUS81*	1.60	x				x	
*SETD2*	1.60	x					x
*ATR*	1.60					x	

*HR, homologous recombination; NHEJ, non-homologous end-joining; BER, base-excision repair; NER, nucleotide-excision repair; ICL repair, interstrand crosslink repair; MMR, mismatch repair.*

The relatively high prevalence of DDR gene mutations opens up opportunities for targeted therapies based on the synthetic lethality principle: tumors with a DDR pathway deficiency are more dependent on alternative DNA repair pathways to repair double-stranded DNA breaks (Guo et al., 2011; Topatana et al., 2020). Synthetic lethality has been applied successfully in cancers harboring *BRCA1/2* mutations (homologous repair pathway) by treating them with *PARP* inhibitors (*PARP* is involved in the single-strand break repair pathway) (Lord and Ashworth, 2017). Unrepaired single-strand breaks will turn into DSBs during DNA replication which will accumulate to the point of cell death due to the HR deficiency.

### DNA Damage Repair Pathways Genomic Profiling/Biomarkers

**FIGURE 2:**
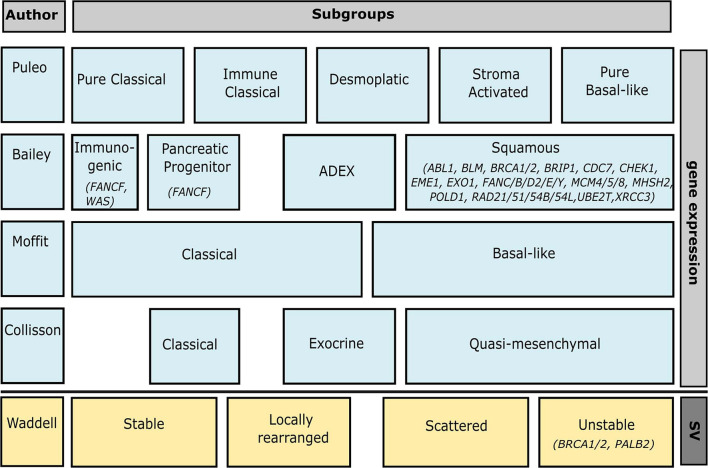
Overview of genomic pancreatic subtypes and how they overlap. Associated DDR genes of which mutations have been found in PDAC patients (see Supplementary Table 1) are included under their respective subtypes. The bar on the right indicates whether the classification is based on gene expression or structural variation (SV). Associated DDR genes not mutated in patients include *CDC45*, *FEN1*, *GINS2/4*, *MAD2L2*, *MCM2/3/6/7*, *RMI2*, *RPA3*, *TIMELESS*, *HMGB2*, *POLA1*, *LIG1*, *DNA2*, *RDC2/3/4/5*, *PCNA*, *COPS5*, *BRIP1*, *HMGA2M*, *CETN2*, *UBC*, *TP73*, *PSMD14*, *POLR2D*, and *CDK7* for Bailey’s squamous subtype, and USP7 for Bailey’s Pancreatic Progenitor subtype. This figure was created in Inkscape.

Multiple research groups have performed next-generation sequencing and expression profiling to classify molecular PDAC subtypes that can be used to tailor therapies and guide clinical decision making (Collisson et al., 2011; Moffitt et al., 2015; Bailey et al., 2016; Puleo et al., 2018). At its simplest, a distinction is made between classical and basal-like subtypes, though most classifications include more specific subtypes as well (Figure 2). Bailey et al. (2016) defined four PDAC subtypes (immunogenic, pancreatic progenitor, ADEX, and squamous) based on 10 discriminatory gene programs found by transcriptional network analysis. Over 50 DDR genes were included in the gene program “proliferation” which is associated with the squamous subtype. Functionally, the squamous subtype is associated with histological adenosquamous carcinoma and a poor survival. The classifications by Collisson et al. (2011), Moffitt et al. (2015), and Puleo et al. (2018) found no associations with DDR deficiency.

Instead of mutational signatures, Waddell et al. (2015) based their classification on structural variation. Using a dataset of 75 primary samples and 25 patient-derived cell lines (PDCLs) they defined four subtypes: stable, locally rearranged, scattered, and unstable. The unstable subtype co-segregated with inactivation of *BRCA1*, *BRCA2*, and *PALB2*, as well as a mutational signature of DDR deficiency. Mutations of *ATM* and other genes involved in DNA maintenance (e.g., *XRCC4/6* and *FANCM*) were also regularly found in these tumors.

Currently only gBRCA is used in the clinic as biomarker for sensitivity to PARP inhibition (PARPi) olaparib. Our query of somatic DDR mutations found that *BRCA2* is mutated in 4% of all PDAC patients indicating that a larger group of patients may benefit from targeted therapy. In addition, multiple clinical trials are recruiting patients for treatment with DDR inhibitors based on a larger selection of DDR mutations, including but not limited to *PALB2*, *CHEK2*, *ATM*, and *RAD51* (Supplementary Table 2). Their outcomes will show whether more DDR genes can be included as biomarkers for targeted therapy in the clinic.

Already, DDR deficiency has been associated with a significantly better patient survival compared to DDR proficiency independently of tumor subtype classification (Zimmermann et al., 2021). However, it should be noted that analysis of the interaction with precise treatment regimens is limited, in particular with regard to receipt of platinum based therapy, and this can be a considerable confounder. In addition, a small retrospective study in 36 patients treated with first-line FOLFIRINOX in a metastatic setting found that DDR deficiency, as based on a 14-gene panel, was significantly associated with improved survival (*p* = 0.04) (Sehdev et al., 2018). While the DDR deficient patient group also had a better median OS (14 vs. 5 months) this difference was not significant (*p* = 0.08). A similar retrospective study in 40 patients with metastatic PDAC treated with first-line platinum chemotherapy in combination with FOLFIRINOX was published a year later (Palacio et al., 2019). Based on a 35-gene panel, the patients with DDR deficiency had a significantly longer progression-free survival (PFS) (18.5 vs. 6.9 months, *p* = 0.003), with a trend toward superior median OS as well. Further research is needed to confirm these findings in a larger cohort and to investigate whether DDR deficiency is associated with response to FOLFIRINOX treatment or OS in general.

### Preclinical Models

Despite the promising results for many targeted therapies in other solid tumors such as breast, lung, and colon, the use of targeted therapies in PDAC has had limited survival benefit in the clinic. Target discovery and successful development of targeted therapies is highly dependent on the relevance of the preclinical models used and therapies frequently fail at the transition to clinical trials. Multiple recent papers are available which review the preclinical models used in PDAC research (Moreira et al., 2018; Garcia et al., 2020; Swayden et al., 2020; Yu et al., 2021). This review will extend upon published literature by focusing on the application of these models to further target DDR pathways.

#### Cell Lines

Cell lines remain the most commonly used preclinical model for cancer research. Their widespread use has ensured that they are readily available and most commercial cell lines are well-characterized (Deer et al., 2010). The main advantages of cell lines are that they are cheap, require little maintenance, and are easy to manipulate. In addition, cell lines are considered to be more homogenous than other preclinical models, thus contributing to a better reproducibility which makes them well-suited for high-throughput drug screening. However, this also means that cell lines lack the complexity and heterogeneity typical of tumors. At the same time, clonality and adaptation to 2D culturing conditions as well as immortalization and repeated passaging can all contribute to genomic drift which can significantly affect drug responses (Hughes et al., 2007; Monberg et al., 2021). Other disadvantages of 2D culture include loss of part of the normal 3D morphology, cell polarity, and cell–cell or cell–stroma interactions, especially the interaction with cancer-associated fibroblasts (CAFs) and immune cells (Feig et al., 2012). While some of these disadvantages can be resolved or diminished by adapting the culture methods (e.g., using early passage PDCLs instead of established cell lines, or co-culturing with fibroblasts) other disadvantages are inherent to the model system itself.

While the possible applications of cancer cell lines are diverse, ranging from biomarker discovery to functional studies, the use of cell lines to study the DDR pathways in PDAC has mainly been limited to drug sensitivity studies. Investigated drugs include PARP inhibitors (e.g., veliparib, olaparib, and rucaparib), WEE1 inhibitors, ATM inhibitors, ATR inhibitors, DNA protein kinase catalytic domain (PRKCD) inhibitors, and more.

DNA damage repair pathway deficiency has been shown to confer sensitivity to PARPi. Multiple studies found that the *BRCA2*-deficient cell line Capan-1 is significantly more sensitive to several PARP inhibitors and cisplatin, but not to gemcitabine, compared to the *BRCA2*-proficient cell lines MiaPaCa-2 and Panc-1 (Porcelli et al., 2013; Andrei et al., 2015; de Soto, 2020). In addition, restoration of *BRCA2* expression in Capan-1 cell lines was shown to reduce sensitivity to olaparib and HYDAMTIQ (Mini et al., 2017; Sullivan-Reed et al., 2018). Similarly, shRNA-mediated knockdown of *BRCA2* in Panc-1 cells impaired homology-directed repair and conferred sensitivity to BMN-673 (but not to veliparib) (Andrei et al., 2015). Furthermore, increased sensitivity to PARPi (olaparib, BMN-673, and rucaparib) and cisplatin has been found in DDR deficient PDCLs (Dreyer et al., 2021).

Acquired resistance is a problem in many cancer treatments. Likewise, long-term treatment of Capan-1 cells with low dose PARPi can induce resistance, including cross-resistance to other PARPi and cisplatin. Several mechanisms have been suggested for the development of resistance in Capan-1 *BRCA2*-deficient cell line, the simplest being the restoration of *BRCA2* expression. Sakai et al. (2008) found that 7 out of 14 Capan-1 clones that developed resistance to cisplatin treatment had additional mutations in the *BRCA2* gene which corrected the original frameshift mutation found in Capan-1. The truncated protein was also still present suggesting that these restorative mutations were preceded by gene duplication. However, the amplification of the truncated protein might in itself also contribute to resistance. Park et al. (2020) investigated PARPi resistant Capan-1 clones and did not detect reversion mutations, though several clones had additional copies of the mutant *BRCA2* allele as well as an increased *BRCA2* protein expression. Immunoprecipitation of *BRCA2* followed by mass spectrometry showed enrichment of *PALB2*, *RAD51*, *MLLT10*, and *DOT1L* in the resistant clones, but not in the parent cells, which may contribute to resistance. Alternatively, Chen et al. (2020) also found additional mutations in *BRCA2* in PARPi resistant Capan-1 clones, but these mutations resulted in truncated splice isoforms. In addition, they found overexpression of the anti-apoptotic proteins *COX-2* and *BIRC3*. Depletion of either *BRCA2*, *COX-2*, or *BIRC3* partially restored PARPi sensitivity. In contrast, combined depletion had no additive effect, suggesting that additional mechanisms contribute to PARPi resistance.

Sensitivity to the *WEE1* inhibitor AZD-1775 has been evaluated in multiple studies, but due to contradicting findings its role in DDR deficiency remains unclear. Two studies found that Capan-1 is markedly more sensitive to AZD-1775 than other (PDAC) cell lines, suggesting that *BRCA2* deficiency might play a role (Dréan et al., 2017; Parsels et al., 2018). However, while restoration of the *BRCA2* open reading frame due to secondary mutations induced by CRISPR-Cas9 reduced the sensitivity to PARP inhibitors olaparib and BMN-673, it did not affect the sensitivity to AZD-1775. On the other hand, Lal et al. (2016) investigated sensitivity to AZD-1775 in a panel of nine PDAC cell lines and reported a medium sensitivity for Capan-1. In addition, they found that knockdown of *BRCA2* by siRNA in MiaPaCa-2 and PL5 induced resistance to AZD-1775. These contradicting findings highlight the need for further investigation.

The application of ATR inhibitors in PDAC has been investigated in multiple *in vitro* studies in both human PDAC cell lines and mouse KPC and KPCB cell lines but so far drugs have shown limited potential and sensitivity to treatment does not correlate with the DDR status (Wallez et al., 2018; Elliott et al., 2019; Dreyer et al., 2021). However, multiple studies have found that ATRi (VE-821 and VE-822) sensitizes to gemcitabine and radiotherapy through impairment of the DNA repair (Fokas et al., 2012; Wallez et al., 2018). siRNA knockdown of another major signal transducer, ATM, in combination with ATRi in MiaPaCa-2 was able to prevent gemcitabine-induced activation of ATR completely (Wallez et al., 2018), suggesting that ATM-mutant tumors may be especially sensitive to this combination treatment. In addition, combination treatment with chloroquine, an autophagy inhibitor that is used in the treatment of malaria, significantly reduced proliferation in 24 or 17 out of 26 tested PDAC cell lines compared to VE-822 or chloroquine alone (Elliott et al., 2019). Azorsa et al. (2009) used an RNAi screen to identify which genes, when silenced, sensitized pancreatic cancer cells to gemcitabine. Silencing of *CHK1* was found to be most effective and was further validated with additional siRNAs and two small molecule inhibitors (SB218078 and PD407824) in MiaPaCa-2 and BxPC3 cell lines (Azorsa et al., 2009).

A disadvantage of DDR targeted therapy is that it is not inherently cytotoxic. By inhibiting multiple DNA repair genes the cancer cells will accrue DNA damage, but whether this results in cell death or senescence depends on additional factors, such as the proliferation rate and how well the cells tolerate replicative stress. Combination treatment with chemo- or radiotherapy can increase the anti-tumor effect by inducing additional DNA damage (Porcelli et al., 2013). Perkhofer et al. (2017) generated stable mouse cell lines from tumors with pancreas-specific loss of *Kras* (KC), and *Kras* and *Atm* (AKC). *Atm*-deficient AKC cells showed a significant increase in DNA damage markers 53BP1 and γH2AFX upon treatment with 5 Gy of ionizing radiation compared to KC cells (*p* < 0.03), indicating impaired DSB repair, and had decreased proliferation. No significant differences were observed in sensitivity to cisplatin, 5-FU, or gemcitabine. Treatment with olaparib or niraparib reduced viability in an *Atm*-dependent manner and was potentiated by combination with gemcitabine or radiation (*p* < 0.01).

The cell lines used for DDR pathway studies in PDAC are mainly limited to Capan-1 as model for DDR/*BRCA2*-deficiency, and MiaPaCa-2, BxPC-3, and Panc-1 for DDR-proficiency. Studies in additional cell line models are required to analyze the role of the DDR pathways in more depth. Table 2 provides an overview of the mutations found in the 10 most frequently mutated DDR genes (excluding *TP53*) as per Table 1 for all PDAC cell lines found in the Cancer Cell Line Encyclopedia. Twenty of the 46 cell lines had a mutation in one or more of the investigated genes, of which three (Capan-1, PL18, and SNU-324) had a mutation annotated as pathogenic or likely pathogenic in the ClinVar or COSMIC database.

**TABLE 2:** Mutation status of DDR genes in cell lines.

Cell line	Gene	Nucleotide change	Protein change	ClinVar	COSMIC FATHMM
BXPC3	*POLQ*		p.ILL1421fs	n/a	n/a
Capan-1	*BRCA2*	c.5946del	p.S1982fs	Pathogenic	n/a
	*ATM*	c.4755A>C	p.R1585S	n/a	n/a
CFPAC1	*PRKDC*	c.1945T>C	p.F649L	Uncertain significance	n/a
HPAC	*NIPBL*		p.T735I	n/a	n/a
HuP-T3	*BRCA2*	c.6131G>T	p.G2044V	Benign/likely benign	n/a
	*NIPBL*		p.1532_1532E>DK	n/a	n/a
KP2	*PRKDC*		p.G2261S	n/a	n/a
	*FAAP100*		p.K333R	n/a	n/a
MZ1PC	*PRKDC*		p.W1355C, p.W1355l, p.F1028V	n/a	n/a
Panc-02.03	*POLQ*		p.L1430fs	n/a	n/a
Panc-03.27	*ATM*	c.7052A>G	p.E2351G	Uncertain significance	n/a
Panc-04.03	*NIPBL*		p.S2389I	n/a	n/a
Panc-08.13	*ATM*		p.F1234S	n/a	n/a
PATU8988S/T	*ATM*		p.R919M	n/a	n/a
PK-45H	*POLQ*		p.G2225R	n/a	n/a
PK-59	*BRCA2*	c.6131G>T	p.G2044V	Benign/likely benign	n/a
PL18	*NIPBL*	c.3G>T	p.M1I	Pathogenic	n/a
			p.S1517*	n/a	n/a
PL4	*RIF1*	c.1331C>T	p.A444V	n/a	Neutral (0.12)
PSN1	*NIPBL*		p.K601fs	n/a	n/a
SNU-324	*BRCA2*	c.7480C>T	p.R2494*	Pathogenic	n/a
	*ATM*		p.Q2809fs	n/a	n/a
	*MCM4*	c.1579G>A	p.V527I	n/a	Pathogenic (0.96)
SW-1990	*NIPBL*		p.K1180*	n/a	n/a
TCCPAN2	*POLQ*		p.R6P	n/a	n/a

Representation of the top 10 (excluding *TP53*) most frequently mutated DDR genes in PDAC cell lines. The genes *WRN* and *FANCD2* were not found to be mutated in any PDAC cell line. When available the pathogenicity status/score is included in the table. The * indicates that the mutation results in an early terminated gene product. n/a, not available.

Apart from Capan-1, SNU-324 is the only other available cell line with a suspected deleterious *BRCA2* mutation (Ku et al., 2002). SNU-324, established in 2001, is derived from a poorly differentiated primary pancreatic tumor of a 50-year-old male. The cells are mainly adherent, but a fraction of the cells grow in suspension and frequently form aggregates. SNU-324 does not contain mutations in *KRAS* or *TP53*, but is microsatellite instable (Ku et al., 2002). Despite its usefulness for *BRCA2*-deficiency studies no other publications are available which have used this cell line. Therefore there is a need for additional well-defined *BRCA2*-deficient PDAC cell lines.

#### Organoids

Patient-derived organoids are still a relatively new model for pancreatic cancer and there are few studies published that focused on the DDR of PDAC organoids. However, there is limited information on patient derived organoid (PDO) sensitivity to DDR-targeted drugs.

Driehuis et al. (2019) performed high-throughput drug screening of 76 drugs in 24 PDOs and found that, in general, PDOs have a similar response to agents that target the same biological process or molecular pathway. Drug response was found to be PDO-specific, thus reflecting patient heterogeneity. Of the 24 PDOs, 1 PDO had a *BRCA2* indel and was among the most sensitive PDOs for most of the tested drugs.

Tiriac et al. (2018) performed pharmacotyping on 66 PDOs for the drugs gemcitabine, paclitaxel, irinotecan, 5-FU, and oxaliplatin. They found that PDO response reflected interpatient variability. For nine patients, the PDO response could be compared to patient response. Eight out of nine patients exhibited an outcome consistent with their matched PDO. Additionally, they investigated the correlation between AUC distribution and genotype for a range of drugs, including olaparib. The three samples with the lowest AUC had *ATM* loss, *PALB2* loss, and *ATM* frameshift plus *BRCA2* loss, respectively. The researchers observed a trend between olaparib sensitivity and complete loss of *PALB2*, but these data must be interpreted cautiously as there were just four PDOs with complete *PALB2* loss. In addition, they state that single-copy losses of a range of genes involved in HR deficiency do not correspond with olaparib sensitivity. In line with these findings Dreyer et al. (2021) reported that they found no correlation between DDR status and the response to *ATR* or *WEE1* inhibition in the six PDOs they investigated.

Based on genomic, transcriptomic, and histologic data, organoids are representative models of PDAC (Tiriac et al., 2018; Driehuis et al., 2019; Gendoo et al., 2019). Yet, limited studies have been published highlighting their use in the study of DDR deficiency in PDAC. However, drug screening and correlation to patient response studies are promising and suggest that organoids are good models to determine drug sensitivity for targeted therapies, and might also be used to identify biomarkers for drug sensitivity.

#### Mouse Models

##### Patient-derived and cell line-derived xenograft models

Patient-derived xenografts (PDXs) are well established cancer models and have been reviewed extensively (Garcia et al., 2020; Shi et al., 2020). PDAC xenografts can be established from resection, biopsy material, and ascites. Copy number alterations and gene expression profiling are largely maintained between primary samples and PDX and genomic signatures can be fitted to the Collisson, Moffitt, and Bailey subtypes (Golan et al., 2017; Nicolle et al., 2017; Pham et al., 2021). Interestingly, even though the mouse hosts are immune-deprived, PDX tumor models can reproduce the immune-related phenotype that is found in certain human primary tumors (Nicolle et al., 2017).

However, the application of PDX toward studying DDR deficiency in PDAC is still used infrequently. Golan et al. (2018) developed six PDXs from metastatic lesions of germline *BRCA*-mutated patients to recapitulate the clinical scenario of *BRCA*-associated PDAC in xenografts. Patient samples were taken before treatment and during progression to represent treatment naïve and resistant patients. Four models had bi-allelic inactivation of *BRCA1/2* and demonstrated increased somatic mutational load compared to the two models that had retained one wild-type copy. Three PDX were treated with olaparib and cisplatin monotherapy, and PDX treatment response as well as HR deficiency profile were found to be associated with patient treatment response to platinum and PARPi.

In a similar study, Lohse et al. (2015) compared treatment sensitivity of four xenografts containing a germline mutation in *BRCA1/2* resulting in heterozygous or homozygous loss to three xenografts with wild-type *BRCA1/2*. Mice were treated for 4 weeks with gemcitabine or cisplatin. The *BRCA* mutant xenografts were significantly more sensitive to both gemcitabine and cisplatin compared to the *BRCA* wild-type xenografts (*p* < 0.0001). In another study, using a *BRCA2* mutant and a *BRCA2* wild-type xenograft, Lohse et al. (2016) found no significant difference in sensitivity to radiation treatment or olaparib. Additionally, olaparib did not sensitize to radiation but instead reduced the induction of DNA damage in the *BRCA* mutant xenografts which was attributed to an increased repair of DSBs by the NHEJ pathway and activation of DNA-PK in the *BRCA* mutant xenograft.

Waddell et al. (2015) compared gemcitabine and cisplatin treatment sensitivity of three PDX with an unstable genome and/or high *BRCA* mutational signature burden to four PDX without. None of the DDR-proficient xenografts responded to cisplatin, while two out of three DDR-deficient xenografts did. The DDR-deficient xenograft that did not respond had a *BRCA* mutational signature burden but no unstable genome or mutation in the *BRCA* pathway. Response to gemcitabine was varied in both groups.

Roger et al. (2020) compared the efficacy of multi-DDR interference as maintenance therapy to continuous FOLFIRINOX treatment in a cell line derived xenograft model. *Atm/Kras*-deficient PDAC mouse cell lines (AKC) were orthotopically transplanted in mice and treated with four cycles of FOLFIRINOX to mimic a clinical setting, followed by either a combination of olaparib, VE-822 (ATRi), and CC-115 (DNA-PKi); FOLFIRINOX; or vehicle until an ethical endpoint was reached. OS was significantly longer in the multi-DDR group compared to the FOLFIRINOX or placebo groups (28.5 vs. 24.5 vs. 18.0 days, *p* < 0.02). In addition, the FOLFIRINOX treatment was shown to select for more aggressive subclones, which could partly be erased by multi-DDR treatment. The combination of multiple targeted drugs allowed for lower dosing than used in monotherapy which reduced side effects to a similar level as for the FOLFIRINOX treatment.

##### Genetically engineered mouse models

The advance of genetic manipulation has allowed for the development of genetically engineered mouse models (GEMMs). Carefully chosen germline mutations induce tumor formation at an early age and at a relatively high penetrance. In contrast to xenografts, tumors in GEMM develop progressively and can therefore also be used to study precancerous lesions and low grade tumors (Gopinathan et al., 2015).

The most used PDAC models are the KC and KPC mice (Hingorani et al., 2003, 2005; Gopinathan et al., 2015; Lee et al., 2016; Ariston Gabriel et al., 2020). The KC mice is characterized by a germline mutation in *Kras* (*K-ras^*LSL.G*12*D/+*^*) and the KPC mice has an additional mutation in *Tp53* (*p53^*LSL.R*172*H/+*^*). The presence of Pdx1-Cre removes the floxed transcriptional STOP cassette which silenced the mutant alleles in the pancreas. Both models develop PanINs and eventually also PDAC although the onset and penetrance of PDAC is later and lower in KC mice.

The KC and KPC mouse models have been instrumental in understanding tumor development in DDR-proficient PDAC but have also been used as a basis for DDR-deficient GEMM models. The following section will describe DDR-deficient GEMM models (*Brca2*-deficient and *Atm*-deficient) that have been published in literature (Table 3).

**TABLE 3:** Overview DDR deficient pancreatic cancer GEMMs studies.

References	Model	Mutations	No. of mice	Phenotype
Hingorani et al. (2003, 2005)	KC	*Pdx1-Cre*; *K-ras^*LSL.G*12*D/+*^*	33	100% developed PanIN which progressed to invasive and metastatic PDAC in a small minority.
	KPC	*Pdx1-Cre*; *K-ras^*LSL.G*12*D/+*^*; *p53^*LSL.R*172*H/+*^*	28	96% developed PDAC which metastasized in over half of the mice. Median survival of 22 weeks.
Feldmann et al. (2011)	CB	*Pdx1-Cre*; *Brca2*^*flox/flox*^	25	15% developed invasive and metastatic PDAC, more mice developed PanIN. Median survival of 65 weeks.
	CBP	*Pdx1-Cre*; *Brca*^*flox/flox*^; *LSL-Trp53^*R*172*H*^*	33	100% developed invasive or metastatic PDAC. Median survival of 54 weeks.
Skoulidis et al. (2010)	CB^*Tr/*Δ11^	*Pdx1*-Cre; *Brca2*^*Tr/*Δ11^	24	No development of pancreatic cancer.
	PCB^*Tr/*Δ11^	*Pdx1-Cre*; *Trp53^*R*270*H*^*; *Brca2^*Tr/*Δ11^*	22	No development of pancreatic cancer.
	PCB^*Tr/WT*^	*Pdx1-Cre*; *Trp53^*R*270*H*^*; *Brca2*^*Tr/WT*^	25	No development of pancreatic cancer.
	KCB^*wt/wt*^	*Pdx1-Cre*; *K-ras^*LSL.G*12*D/+*^*	40	15% developed PDAC.
	KCB^*Tr/wt*^	*Pdx1-Cre*; *K-ras^*LSL.G*12*D/+*^*; *Brca2*^*Tr/wt*^	40	30% developed PDAC.
	KCB^*Tr/*Δ11^	*Pdx1-Cre*; *K-ras^*LSL.G*12*D/+*^*; *Brca2*^*Tr/*Δ^*^11^*	32	19% developed PDAC, though frequent development of pancreatic insufficiency.
	KPCB^*wt/wt*^	*Pdx1-Cre*; *K-ras^*LSL.G*12*D/+*^*; *p53^*LSL.R*172*H/+*^*	30	80% developed PDAC. Median PDAC-free survival 24 weeks.
	KPCB^*Tr/*Δ11^	*Pdx1-Cre*; *K-ras^*LSL.G*12*D/+*^*; *p53^*LSL.R*172*H/+*^*; *Brca2*^*Tr/*Δ^*^11^*	30	87% developed PDAC. Median PDAC-free survival 12 weeks.
	KPCB^*Tr/wt*^	*Pdx1-Cre*; *K-ras^*LSL.G*12*D/+*^*; *p53^*LSL.R*172*H/+*^*; *Brca2*^*Tr/wt*^	30	97% developed PDAC. Median PDAC-free survival 20 weeks.
Rowley et al. (2011)	CB2*^Δ11/Δ11^*	*Pdx1-Cre*; *Brca2^Δ11/Δ11^*	12	No development of precursor lesions or PDAC.
	CB2^*wt/*Δ11^	*Pdx1-Cre*; *Brca2^*wt/*Δ11^*	21	No development of precursor lesions or PDAC.
	CPB2*^Δ11/Δ11^*	*Pdx1-Cre*; *Trp53^*F*2–10/F2–10^*; *Brca^Δ11/Δ11^*	34	High frequency development of pancreatic cancer, >40% of ductal origin
	CPB2*^*wt/*Δ11^*	*Pdx1-Cre*; *Trp53^*F*2–10/F2–10^*; *Brca^*wt/*Δ11^*	41	Development of pancreatic cancer, >40 of ductal origin.
	CPB2*^*wt/wt*^*	*Pdx1-Cre*; *Trp53^*F*2–10/F2–10^*	47	Development of pancreatic cancer, predominantly acinar, and undifferentiated.
Russell et al. (2015)	AKC^±^	*Pdx1-Cre*; *K-ras^*LSL.G*12*D/+*^*, *Atm*^*flox/+*^	32	Development of PanIN. Median survival 36 weeks.
	AKC^–/–^	*Pdx1-Cre*; *K-ras^*LSL.G*12*D/+*^*, *Atm*^*flox/flox*^	15	Development of PanIN. Median survival 45 weeks.
Drosos et al. (2017)	KC	*Pdx1-Cre*; *K-ras^*LSL.G*12*D/+*^*, *Ptf1a*^+/cre^	19	42% developed pancreatic cancer of which >80 of sarcomatoid histology, median survival 61 weeks.
	KCATMΔ+	*Pdx1-Cre*; *K-ras^*LSL.G*12*D/+*^*, *Ptf1a*^+/cre^, *Atm*^*loxP/+*^	21	62% developed pancreatic cancer mainly poor and moderately differentiated, median survival 39 weeks.
	KCATMΔΔ	*Pdx1-Cre*; *K-ras^*LSL.G*12*D/+*^*, *Ptf1a*^+/cre^, *Atm*^*loxP/loxP*^	18	94% developed pancreatic cancer with a mixture of moderate, poor, and undifferentiated tumors, median survival 39 weeks.

##### *Brca2*-deficient genetically engineered mouse model

Feldmann et al. (2011) established two *BRCA2*-mutated GEMM, abbreviated as CB (*Pdx1-Cre*; *Brca2*^*flox/flox*^) and CBP (*Pdx1-Cre*; *Brca*^*flox/flox*^; *LSL-Trp53^*R*172*H*^*), and performed extensive histopathological characterization and survival analysis. Both models developed the full spectrum of PanIN lesions which replaced the pancreatic parenchyma and acinar tissue. The additional *Trp53* mutation in the CBP cohort enhanced the frequency of invasive neoplasia and resulted in earlier mortality (375 vs. 454 days, *p* = 0.085). Five mice from the CBP cohort developed metastatic lesions (after 15 months), two were categorized as moderately differentiated adenocarcinomas, two as a combination of adenocarcinoma and sarcomatoid carcinoma, and one as anaplastic carcinoma with giant cells. Sequencing of metastatic PanIN (*n* = 2) and PDAC (*n* = 2) in CB and CBP mice found no secondary *Kras* mutations in any of the mutation hotspots (codon 12, 13, and 61) indicating that *Kras* mutation is not prerequisite for tumorigenesis in the presence of *Brca2* mutation in mice.

These findings were contradicted by Skoulidis et al. (2010) who argued that *Brca2*-deficiency alone is not sufficient to induce carcinogenesis. They developed several mouse models with a combination of mutations in *Brca2*, *Trp53*, and *Kras* (Skoulidis et al., 2010). Of the resulting models, those with solely *Brca2*-deficiency (CB^*Tr/*Δ11^: *Pdx1*-Cre; *Brca2*^*Tr/*Δ11^) had a longer survival than those with combined *Brca2*-deficiency and *Trp53* loss (PCB^*Tr/*Δ11^: *Trp53^*R*270*H*^*, *Pdx1-Cre*; *Brca2^*Tr/*Δ11^* and PCB^*Tr/WT*^: *Trp53^*R*270*H*^*, *Pdx1-Cre*; *Brca2*^*Tr/WT*^) which the researchers contribute to development of pancreatic insufficiency in a fraction of mice. Mice with triple mutation (KPCB^*Tr/*Δ11^: KPC mouse with additional *Pdx1*-Cre; *Brca2*^*Tr/*Δ11^) nearly all developed tumors and had the worst survival. However, homozygous *Brca2* inactivation did contribute to a significantly more aggressive disease with rapid clinical decline compared to wild-type or heterozygous loss in combination with *Kras* and *Trp53* mutation (*p* < 0.002). Tumors in KCB and KPCB mice displayed a range of histological features that can also be found in human pancreatic cancers, ranging from PDAC to sarcomatoid tumors and acinar-cell carcinoma.

In line with this Rowley et al. (2011) found that loss of *Brca2* alone is not enough to induce tumorigenesis, but that it can promote tumorigenesis in combination with *Trp53* inactivation. They developed *Brca2*-deficient (CB2*^Δ11/Δ11^*: *Pdx1-Cre*; *Brca2^Δ11/Δ11^*; CB2^*wt/*Δ11^: *Pdx1-Cre*; *Brca2^*wt/*Δ11^*) and *Trp53*-null (CPB2*^Δ11/Δ11^*, CPB2*^*wt/*Δ11^*, and CPB2*^*wt/wt*^*) mice. The CB2 mice did not develop PDAC whereas the CPB2 mice did. Heterozygous and homozygous *Brca2* loss significantly reduced pancreatic cancer-free survival (*p* < 0.0001), with the strongest effect seen in the homozygous-loss mice. The tumors observed in these mice were similar to several human pancreatic cancer types: 40% were of ductal origin, 35% high grade undifferentiated carcinomas, 20% were mucinous tumors, and the remaining 15% were acinar carcinomas. CPB2*^*wt/wt*^* mice presented mainly with acinar and undifferentiated tumors. Seventy-two percent of the CPB2*^Δ11/Δ11^* mice were found to have PanIN lesions at the time of tumor resection or death, while this was less than 6% in CPB2*^*wt/*Δ11^* and CPB2*^*wt/wt*^* mice. Remarkably, additional *Kras^*G*12*D*^* mutation (CKB2 mice) decreased pancreatic cancer formation; Tumors were found in 66% of CKB2*^*wt/*Δ11^* and 61% of CKB2*^*wt/wt*^* mice, but in just 13% of CKB2*^Δ11/Δ11^* mice. The majority of these tumors (>90%) were PDACs.

These studies established that bi-allelic loss of *Brca2* in combination with *Tp53* deregulation can induce a spectrum of pancreatic lesions. Whether bi-allelic loss of *Brca2* alone can also induce pancreatic cancer remains unclear as Feldmann et al. (2011) did not investigate the mutation status of other genes besides *Kras* and *Tp53*.

##### *Atm*-deficient genetically engineered mouse model

Two studies have published a KC *Atm*-deficient mouse model. Russell et al. (2015) found that KC mice with floxed *Atm* (abbreviated as AKC) had developed more acinar-to-ductal metaplasia lesions and PanINs compared with KC mice at 10 weeks old. The higher tumorigenicity of KC *Atm*-deficient mice was confirmed by Drosos et al. (2017) who studied KC mice with either *Atm*^*loxP/+*^ or *Atm*^*loxP/loxP*^ (abbreviated as KCATMΔ+ and KCATMΔΔ). Post-mortem analysis identified pancreatic cancers in 94 and 62% of the KCATMΔ+ and KCATMΔΔ mice compared to 42% in KC mice. In addition, the *Atm*-deficient mice had a comparable and significantly reduced median OS in both studies (*p* < 0.01).

Both studies performed subtype analysis. Russell et al. (2015) performed gene expression profiling of 10-week-old KC and AKC mice and compared this to PDAC subtypes as described by Collisson et al. (2011). Hierarchical clustering revealed that AKC pancreatic tumors were closer associated with the quasi-mesenchymal human PDAC subtype than with KC pancreatic tumors. In contrast, Drosos et al. (2017) used *KC*, *KCATMΔ*+ and *KCATMΔΔ* primary tumor cell lines for subtyping and concluded that their tumors were primarily of the pancreatic progenitor/classical phenotype based on the high expression of several progenitor marker genes (*Pdx1*, *Hnf1β*, and *Lgals4*) and a classical marker gene (*Gata6*). This discrepancy in subtyping may be explained by the sample material used. The subtyping as defined by Collisson et al. (2011) is based on FFPE material and therefore includes tumor cells, stroma, and normal tissue, by using cell lines the gene expression profile is altered compared to the primary tissue due to the lack of stroma which will likely affect the subtype definition, especially with respect to the quasi-mesenchymal subtype.

### Clinical Trials Targeting DNA Damage Repair Deficiency Pathways

The past decade has seen a rising interest in the combination of cytotoxic chemotherapy with targeted approaches to exploit the synthetic lethality of this combinatorial approach. As of July 2021, there are 51 clinical trials registered on clinicaltrials.gov that investigate DDR targeted therapies alone or in combination with chemotherapy in PDAC (either in PDAC alone or as part of a larger cancer patient cohort). The majority of these trials (78%, *n* = 40) focus on *PARP* inhibitors, although *ATM/ATR*, *CHK1*, *DNA-PK*, and *WEE1* inhibitors are also being investigated (Supplementary Table 2).^1^ Except for a single trial, all trials are either phase I or II, with limited numbers of patients and often single-arm treatment protocols which renders efficacy analysis more challenging.

#### PARP Inhibitors

The pivotal phase III trial leading to FDA approval for the use of PARPi in metastatic PDAC was conducted by Golan et al. (2019) and evaluated olaparib as maintenance therapy in metastatic PDAC patients with germline mutation of *BRCA* (NCT02184195). Patients were eligible if their tumor had not progressed on first-line platinum-based chemotherapy (e.g., cisplatin or oxaliplatin). Treatment with olaparib was compared to placebo and a significant increase in PFS was observed (7.4 vs. 3.8 months, *p* = 0.004), but at interim analysis (data maturity 46%) no significant difference was found in median OS (18.9 vs. 18.1 months, *p* = 0.68). The updated results of this study were presented in January 2021 (Golan et al., 2021). Disappointingly there was again no difference in median OS between the groups (OS 19.0 vs. 19.2 months, *p* = 0.35). Notably, however, PFS2, i.e., the time from randomization to second disease progression or death, was significantly longer in the olaparib-treated group (PFS2, 16.9 vs. 9.3 months, *p* = 0.0061).

A comparable phase I trial (NCT00515866) in PDAC patients with locally advanced or metastatic PDAC being treated in the first line setting included a comparison of olaparib combined with gemcitabine vs. gemcitabine alone in the expansion phase (*n* = 22) (Bendell et al., 2015). Patients were eligible for inclusion regardless of genetic/molecular status. No significant benefit was found regarding objective response rate (ORR), OS, or PFS for the combination treatment. While the researchers noted that nine patients had *BRCA* mutation status available, analysis of response by *BRCA* mutation status was not performed due to the small number of patients for whom this data was recorded.

O’Reilly et al.’s (2018) phase IB trial of the addition of another PARPi, veliparib, to first line chemotherapy (cisplatin, gemcitabine) demonstrated a striking ORR of 78% in patients with stage III/IV PDAC with *BRCA1/2* germline mutations and an equally impressive median OS of 23.3 months. The investigators then proceeded to a phase II trial of this combination in patients with PDAC and germline *BRCA* or *PALB2* mutations (O’Reilly et al., 2020). Response rate in the combination arm was 79 vs. 65.2% in the chemotherapy alone arm (*p* = 0.02). However, there was no statistically significant difference in PFS or OS between the groups (PFS 10.1 vs. 9.7 months, *p* = 0.73; OS 15.5 vs. 16.4 months, *p* = 0.6). The 2- and 3-year OS of 30.7 and 17.8%, respectively, in this study are the longest ever reported in a clinical trial in this cohort. Notably a phase II study of veliparib alone in the second line setting in patients with *BRCA* mutant PDAC reported no confirmed responses; 4 patients (4/16) had stable disease for 4 months (Lowery et al., 2018).

Two additional phase II trials have published results on the addition of veliparib to 5-FU based chemotherapy. The first study (NCT02890355) compared the combination of veliparib with modified FOLFIRI vs. FOLFIRI alone as second line treatment in metastatic pancreatic cancer patients (Chiorean et al., 2019). In total, 108 patients were included in the analysis. The addition of veliparib to FOLFIRI treatment was shown to increase toxicity. Moreover, veliparib did not improve either OS (5.1 vs. 5.9 months in combination vs. monotherapy arm, respectively, HR 1.3, *p* = 0.21) or PFS (2.1 vs. 2.9 months, HR 1.5, *p* = 0.05). Additionally, blood and tumor biopsies were collected at baseline to explore HR or DDR biomarkers. Nine percent of the tumors had HR deficiency (*BRCA1/2*, *ATM*, *PALB2*, *ATM*, or *CDK12* mutation), and an additional 20% had mutations in other DDR genes (*FANC*, *BLM*, *SLX4*, *CHEK2*, *POLD1*, *RIF1*, and *MSH2/6*). Correlative analysis of HR or DDR deficiency with treatment response is still ongoing.

Pishvaian et al. (2020) performed a phase I/II clinical trial (NCT01489865) to evaluate the safety and response of PDAC patients to combination treatment of veliparib with modified FOLFOX. For the phase I portion of the study patients were not selected based on genetic history; however, for the phase II part of the trial, patients were selected based on the presence of HR-DDR deficiency or family history suggesting breast or ovarian cancer syndrome, and a distinction was made between previously treated and untreated patients. The ORR was 20% in the phase I unselected cohort (*n* = 23) and 31% in the phase II cohort (*n* = 33) selected for HR-DDR deficiency. Further analysis of the phase II cohort showed that treatment-naïve patients had a better ORR and OS than previously treated patients (40 vs. 22%, and 13.0 vs. 4.5 months, respectively). In the treatment-naïve HR-DDR patients, the ORR was 57%. However, due to the lack of a placebo or control arm the magnitude of benefit attributable to the addition of veliparib is difficult to quantify. Previously reported OS of metastatic PDAC patients receiving FOLFOX as second-line treatment are in a similar range (3.3 and 6.3 months) (Yoo et al., 2009; Hecht et al., 2020).

Multiple trials for PARP inhibitors are currently recruiting or preparing for recruitment of patients with DDR deficiency (either for *BRCA* mutations specifically, or for a panel of DDR genes). This includes phase I and II trials for niraparib both as monotherapy and in combination with other drugs (NCT04673448, NCT04764084, NCT03601923, and NCT04493060), Olaparib (NCT04548752), rucaparib (NCT04171700), talazoparib (NCT04550494), and NMS-03305293 (NCT04182516).

#### CHK1 Inhibitor

Laquente et al. (2017) performed a phase I/II clinical trial for the *CHK1* inhibitor rabusertib (LY2603618) combined with gemcitabine vs. gemcitabine alone in patients with locally advanced or metastatic PDAC (NCT00839332). Although no significant differences were found in the number and severity of adverse events, no significant differences in OS, PFS, ORR, or duration of response were found either.

#### DNA-PK Inhibitor

A phase II trial on the safety and efficacy of the combination of the *DNA-PK* inhibitor LY3023414 with abemaciclib in previously treated metastatic PDAC patients compared to standard-of-care gemcitabine or capecitabine found that the combination treatment had a significantly worse PFS (1.81 vs. 3.25 months, *p* = 0.012) (NCT02981342).

#### Patient Selection

Preclinical models have shown that several targeted therapies are more effective in models that defects in complementary DNA repair pathways and this principle of synthetic lethality has been adapted by clinical trials. Multiple trials are currently running which select patients based on the presence *BRCA* mutations. While the application of targeted therapy in patients with of *BRCA* mutations has had success in other cancer types (most notably in breast and ovarian cancer), the fraction of patients with *BRCA* mutations is relatively small and patients with other DDR gene mutations may also benefit from these therapies. Several studies have included additional mutations in their selection criteria, but based on Table 1 these panels could be extended upon. However, preclinical trials might be needed to warrant this.

Alternatively, DDR deficiency might serve as biomarker for response to non-targeted therapies. One study that is currently investigating this is the Precision Panc trial which recruits PDAC patients for molecular profiling and allows patients to enroll in a PRIMUS trial (NCT0461417), one of which aims to test a DDR deficiency biomarker for response to FOLFOX-A treatment (NCT04176952).

## Discussion

The dismal survival rate of PDAC underscores the urgent need to develop new and more effective preventative and therapeutic strategies. So far, clinical trials for DDR targeting drugs have shown limited results beyond the approval of Olaparib in the maintenance setting for patients with germline *BRCA1/2* mutations. To improve treatment, representative models are needed, especially those that model DDR deficiency, to test drugs and to develop biomarkers that predict patient response. Clinical rationale exists to expand the use of DDR targeting agents, however, to date there are no validated predictors of treatment response with these agents for patients with DDR deficient tumors beyond *BRCA1/2*.

Therefore, in order to expand the impact of targeting DDR pathway genes, we performed a comprehensive review of published preclinical models which can potentially be used for DDR deficiency targeted drug screening. Using a 352 DDR gene panel we queried the GENIE database and found multiple frequently mutated DDR genes, some of which are not commonly used in DDR panels. This suggests that DDR deficiency might occur more frequently than previously thought but also provides additional options for biomarkers or targeted therapies.

Cell lines are by far the most frequently used model for PDAC. However, DDR deficiency studies are mainly limited to the *BRCA2*-mutant cell line Capan-1. Our query of the PDAC cell lines in the CCLE database for the top 10 most commonly mutated DDR genes (excluding *TP53*) found that 20 cell lines contain one or multiple DDR gene mutations. However, for the majority of these mutations the pathogenicity is unknown and functional characterization of the DDR status is warranted.

The application of organoids toward PDAC studies is still relatively new and at the time of writing no studies in PDAC organoids on DDR-deficiency specifically have been published. We therefore stress the need for characterization of DDR status in existing organoids based on NGS and functional profiling as well as the development and characterization of DDR deficiency in existing organoids through gene editing.

In contrast to cell lines and organoids, several DDR deficient mouse models have been published. So far the application of these models has mainly focused on the contribution of DDR deficiency on tumorigenesis and tumor progression, but there is opportunity for their application in drug sensitivity studies.

The process from biomarker/drug discovery to clinical practice is a long and often unsuccessful path, with 95% of the drugs failing at the translation from preclinical model to clinical trials (Seyhan, 2019). Preclinical models that closely recapitulate the primary tumor have a higher translational value and thus improve the chance of success.

Interest in DDR deficiency as a target for personalized therapy is rising, indicated by the high number of clinical trials currently open in this area. Of the 51 trials running, 29 phase I or II trials are currently recruiting or not yet recruiting. More evidence of the efficacy of these therapies in preclinical PDAC models are required to support their clinical rationale, as many studies have not achieved their desired endpoint. While limitations exist to preclinical *in vitro* and *in vivo* models (Nelson and Walsh, 2020), appropriate preclinical models can reflect histopathological subtypes, assist in early prioritization of promising therapies, be used in high-throughput screening to identify ineffective therapies earlier, and thus prevent the excessive time and money resources of a failed clinical trial.

## Author Contributions

NW and ML conceived the study and provided the significant guidance. NW drew up the outline of the review. JS wrote the manuscript and generated the illustrations. EH contributed with the section on clinical trials. SM contributed to the writing of early drafts. All authors approved the final manuscript.

## Conflict of Interest

The authors declare that the research was conducted in the absence of any commercial or financial relationships that could be construed as a potential conflict of interest.

## Publisher’s Note

All claims expressed in this article are solely those of the authors and do not necessarily represent those of their affiliated organizations, or those of the publisher, the editors and the reviewers. Any product that may be evaluated in this article, or claim that may be made by its manufacturer, is not guaranteed or endorsed by the publisher.

## Footnotes

1 The status of the trials is not always up-to-date, several trials have been completed and published the results but are still marked as “active.”
