# Supplementary material for: DNA Damage Repair Deficiency in Pancreatic Ductal Adenocarcinoma: Preclinical Models and Clinical Perspectives
Source: Front Cell Dev Biol. 2021 Oct 12;9:749490. doi: 10.3389/fcell.2021.749490 (PMC8546202; doi:10.3389/fcell.2021.749490)
Supplement: Supplementary file 1 [file Table_1.docx]

**Supplemental Table 1. Mutation frequency of DDR genes in PDAC patients and their associated pathways.** Abbreviations: HR: homologous recombination, NHEJ: non-homologous end-joining, BER: base-excision repair, NER: nucleotide-excision repair, ICL repair: interstrand crosslink repair, MMR: mismatch repair.

| **Gene** | **Freq (%)** | **Pathways** |
| --- | --- | --- |
| *TP53* | 68.90 | NER |
| *BRCA2* | 4.40 | HR, NER |
| *ATM* | 4.00 | NHEJ |
| *PRKDC* | 3.90 | NHEJ |
| *MCM4* | 3.50 | HR |
| *NIPBL* | 3.20 | HR |
| *POLQ* | 3.20 | HR, NHEJ, BER |
| *RIF1* | 3.10 | HR, NHEJ |
| *WRN* | 2.40 | HR, BER |
| *FAAP100* | 2.40 | ICL repair |
| *FANCD2* | 2.40 | ICL repair |
| *ERCC6* | 2.20 | HR, NHEJ, BER, NER |
| *EP300* | 2.10 | NER |
| *RECQL4* | 2.00 | HR |
| *HELQ* | 1.90 | HR |
| *CUL4A* | 1.80 | NER |
| *ARID2* | 1.80 | HR |
| *FANCM* | 1.80 | HR, ICL repair |
| *FANCA* | 1.80 | ICL repair |
| *PAXIP1* | 1.70 | NHEJ |
| *FAN1* | 1.60 | HR, NER, ICL repair |
| *BRCA1* | 1.60 | HR, NHEJ |
| *MUS81* | 1.60 | HR, ICL repair |
| *SETD2* | 1.60 | HR, MMR |
| *ATR* | 1.60 | ICL repair |
| *SLX4* | 1.40 | HR, NER, ICL repair |
| *RAD54B* | 1.40 | HR |
| *BRCC3* | 1.40 | NHEJ |
| *MSH6* | 1.40 | ICL repair, MMR |
| *POLE* | 1.30 | BER, NER |
| *GEN1* | 1.30 | HR |
| *PALB2* | 1.30 | HR |
| *XRCC3* | 1.30 | HR, ICL repair |
| *UIMC1* | 1.30 | NHEJ |
| *XPC* | 1.20 | NER, MMR |
| *MCM8* | 1.20 | HR, ICL repair |
| *RBBP8* | 1.20 | HR |
| *TP53BP1* | 1.20 | HR, NHEJ |
| *NEIL3* | 1.20 | BER, ICL repair |
| *XRCC1* | 1.10 | HR, NHEJ, BER, NER |
| *BLM* | 1.10 | HR |
| *POLD1* | 1.00 | BER, NER, MMR |
| *AXIN2* | 1.00 | MMR |
| *DCLRE1C* | 1.00 | NHEJ, ICL repair |
| *FANCI* | 1.00 | ICL repair |
| *NEIL2* | 1.00 | BER |
| *ERCC5* | 0.90 | HR, BER, NER |
| *LIG4* | 0.90 | NHEJ, NER |
| *CDC7* | 0.90 | HR |
| *MCM5* | 0.90 | HR |
| *RAD50* | 0.90 | HR, NHEJ |
| *ABL1* | 0.90 | MMR |
| *BARD1* | 0.90 | NHEJ |
| *EME1* | 0.90 | ICL repair |
| *MBD4* | 0.90 | BER |
| *MLH3* | 0.90 | MMR |
| *MSH2* | 0.90 | MMR |
| *BRIP1* | 0.80 | NER |
| *ERCC2* | 0.80 | NER |
| *FUS* | 0.80 | HR |
| *NBN* | 0.80 | HR, NHEJ |
| *MDC1* | 0.80 | NHEJ |
| *XPA* | 0.70 | BER, NER, ICL repair |
| *FANCL* | 0.70 | ICL repair |
| *MLH1* | 0.70 | NHEJ, MMR |
| *NEIL1* | 0.70 | BER |
| *NSD2* | 0.70 | NHEJ |
| *TDG* | 0.70 | BER, MMR |
| *XRCC6 (Ku70)* | 0.70 | NHEJ |
| *ERCC4* | 0.60 | HR, NHEJ, NER, ICL repair |
| *ERCC1* | 0.60 | NHEJ, NER, ICL repair, MMR |
| *FANCC* | 0.60 | NER, ICL repair |
| *WAS* | 0.60 | HR |
| *FANCE* | 0.60 | ICL repair |
| *FANCG* | 0.60 | ICL repair |
| *MUTYH* | 0.60 | BER, MMR |
| *PARP3* | 0.60 | NHEJ |
| *PMS2* | 0.60 | MMR |
| *DDB2* | 0.50 | NER |
| *OGG1* | 0.50 | BER, NER |
| *RAD21* | 0.50 | HR |
| *EXO1* | 0.50 | MMR |
| *FANCF* | 0.50 | ICL repair |
| *MSH3* | 0.50 | MMR |
| *POLB* | 0.50 | NHEJ, BER |
| *RNF8* | 0.50 | NHEJ, ICL repair |
| *UBE2T* | 0.50 | ICL repair |
| *XRCC4* | 0.50 | NHEJ |
| *XRCC5 (Ku80)* | 0.50 | NHEJ |
| *PARP1* | 0.40 | HR, BER, NER |
| *RPA1* | 0.40 | HR, BER, NER, ICL repair, MMR |
| *ERCC3* | 0.40 | NER |
| *CHEK1* | 0.40 | HR |
| *FANCB* | 0.40 | HR, ICL repair |
| *ABRAXAS1* | 0.40 | NHEJ |
| *PMS1* | 0.40 | MMR |
| *POT1* | 0.40 | NHEJ, BER |
| *RAD52* | 0.30 | HR, NER |
| *NTHL1* | 0.30 | BER, NER |
| *PNKP* | 0.30 | NHEJ, BER, NER |
| *DMC1* | 0.30 | HR |
| *RAD51C* | 0.30 | HR |
| *RAD54L* | 0.30 | HR |
| *SLX1A* | 0.30 | HR, ICL repair |
| *BABAM2* | 0.30 | NHEJ |
| *FAAP24* | 0.30 | ICL repair |
| *DDB1* | 0.20 | NER |
| *MRE11* | 0.20 | HR, NHEJ |
| *RAD51* | 0.20 | HR, ICL repair |
| *RECQL* | 0.20 | HR |
| *RTEL1* | 0.20 | HR |
| *BABAM1* | 0.20 | NHEJ |
| *FAAP20* | 0.20 | ICL repair |
| *XRCC2* | 0.10 | HR |
| *PPP4R2* | 0.09 | HR |
| *RAD51B* | 0.09 | HR |
| *RAD51D* | 0.09 | HR, ICL repair |
