# Supplementary material for: DNA Damage Repair Deficiency in Pancreatic Ductal Adenocarcinoma: Preclinical Models and Clinical Perspectives
Source: Front Cell Dev Biol. 2021 Oct 12;9:749490. doi: 10.3389/fcell.2021.749490 (PMC8546202; doi:10.3389/fcell.2021.749490)
Supplement: Supplementary file 2 [file Table_2.DOCX]

**Supplemental table 2**. Overview of clinical trials in PDAC that use DDR targeting therapies (clinicaltrials.gov). Abbreviations: OS: overall survival, ORR: overall response rate, OR: odds ratio, HR: hazard ratio, AE: adverse event, SAE: serious adverse event, DL: dose level, CBR: clinical benefit rate, DCO: data cut-off

| **NCT ID (publication)** | **Phase** | **Drug** | **Condition** | **Intervention** | **Status** | **Results (months, CI)** |
| --- | --- | --- | --- | --- | --- | --- |
| **ATR** | | | | | | |
| NCT01337765 | 1 | Dactolisib | advanced non resectable solid tumors | Dactolisib, MEK162 | completed |  |
| NCT04514497 | 1 | Elimusertib | metastatic or unresectable SCLC, PDNEC, PDAC | Arm A: limusertib, irinotecan liposome  Arm B: elimusertib, topotecan | not yet recruiting |  |
| NCT04616534 | 1 | Elimusertib | uncurable solid tumor | Gemcitabine, elimusertib | not yet recruiting |  |
| NCT04657068 | 1/2 | ART0380 | advanced or metastatic solid tumors | Part A1: intermittent vs. continuous ART0380  Part A2: intermittent ART0380, gemcitabine  Part B1: ART0380 in patients without *ATM* mutations  Part B2: ART0380, gemcitabine vs. gemcitabine in patients with advanced ovarian cancer | recruiting |  |
| **DNA-PK** | | | | | | |
| NCT02981342 | 2 | LY3023414 | metastatic PDAC | Arm A: abemaciclib  Arm B: abemaciclib, LY3023414  Arm C: SOC (gemcitabine/capecitabine) | completed | PFS abemaciclib: 1.68 (1.35 to 1.84)  abemaciclib+LY3023414: 1.81 (1.28 to 1.91)  SOC: 3.25 (1.05 to 5.65) |
| NCT04172532 | 1/2 | Peposertib | locally advanced PDAC | Phase 1: hypofractionated radiation, M3814  Phase 2, arm A: hypofractionated radiation, M3814  Phase 2, arm B: hypofractionated radiation, placebo | recruiting |  |
| **PARP** | | | | | | |
| NCT02184195 (Golan et al., 2019) | 3 | Olaparib | PDAC (*BRCA*-mut) | Arm A: olaparib  Arm B: placebo | active, not recruiting | PFS olaparib: 7.4 (4.14 to 11.01) vs. placebo: 3.8 (3.52 to 4.86), p=0.004  mOS Olaparib: 18.9 (14.85-26.15) vs. placebo 18.1 (12.62-26.12), p=0.6833 |
| NCT03205176 | 1 | Olaparib | solid tumors | Arm A: AZD5153 (BRD4/BETi)  Arm B: AZD5153, olaparib | completed | Preliminary safety data: 50% AEs, 25% SAEs, 25%≥G3 AEs |
| NCT02677038 | 2 | Olaparib | metastatic PDAC post ≥ first-line of prior therapy | Olaparib | active, not recruiting |  |
| NCT03140670 | 2 | Rucaparib | locally advanced or metastatic PDAC with *BRCA1/2* or *PALB2* mut that has not progressed on platinum-based therapy | Maintenance rucaparib | active, not recruiting |  |
| NCT02890355 (Chiorean et al., 2019) | 2 | Veliparib | metastatic PDAC | Arm A: veliparib, mFOLFIRI  Arm B: FOLFIRI | active, not recruiting | OS veliparib+mFOLFIRI: 5.4 (3.7 to 7.2)  mFOLFIRI: 6.5 (5.6 to 7.8), p=0.28 |
| NCT01489865 (Pishvaian et al., 2020) | 1/2 | Veliparib | PDAC (BRCAness) | Veliparib, mFOLFOX-6 | active | mOS all patients: 8.5, mOS platinum-naïve (DDR mut): 11.8  ORR entire cohort: 26%  ORR in platinum-naïve (DDR mut): 57% |
| NCT01585805 | 1 | Veliparib | advanced/metastatic PDAC (*BRCA/PALB2* mut) | Arm A: veliparib, gemcitabine HCl, cisplatin  Arm B: gemcitabine HCl, cisplatin  Arm C: veliparib | active, not recruiting | phase I result (n=17)  OR: BRCA+ 7/9, BRCA- 0/7.  OS: BRCA+ 23.3, BRCA- 11 |
| NCT01296763 | 1/2 | Olaparib | PDAC | Irinotecan, cisplatin, olaparib (mitomycin C) | completed | severe toxicity, further study not planned. SAEs 71.4% at DL1, 80% at DL2, 66,7% at DL3 |
| NCT00515866 (Bendell et al., 2015) | 1 | Olaparib | Locally advanced or metastatic unresectable PDAC | Dose escalation phase: olaparib, gemcitabine  Dose expansion: olaparib, gemcitabine vs. gemcitabine | completed | ORR expansion phase: olaparib + gemcitabine: 27% gemcitabine:14%, p=ns  (no sign. difference OS or PFS) |
| NCT02042378 | 2 | Rucaparib | PDAC (*BRCA* mut, germline or somatic) | Rucaparib | completed | ORR: 15.8%, DCR: 31.6 |
| NCT01286987 | 1 | Talazoparib | unresectable, locally advanced or metastatic solid tumor | Talazoparib | completed | PaCa PFS: 5.3 weeks (2.4 to 21.3) |
| NCT02286687 | 2 | Talazoparib | Metastatic or inoperable locally advanced or recurrent cancer with somatic mutations or deletions in *BRCA1/2,* mutations or homozygous deletions in other *BRCA* pathway genes, or germline *BRCA1/2* mutations with cancers other than breast or ovarian | Talazoparib | recruiting |  |
| NCT00892736 | 1 | Veliparib | malignant solid tumors with *BRCA1/2* mutations, platinum refractory ovarian, fallopian tube or primary peritoneal cancer, or progressive basal-like breast cancer | Veliparib | completed | *BRCA* mut: ORR 23%, CBR of 58%  *BRCA* wt: ORR 3%, CBR of 38% |
| NCT01908478 | 1 | Veliparib | advanced unresectable or borderline resectable PDAC | Veliparib, gemcitabine, radiation | completed | mOS entire cohort: 14.6 (11.6-21.8)  mOS DDR gene altered cohort: 19 (6.2-27.2) |
| NCT04673448 | 1 | Niraparib | uresectable or metastatic breast, pancreas, ovary, fallopian tube or primary peritoneal cancer with *BRCA* mut | Niraparib, dostarlimab | not yet recruiting |  |
| NCT04764084 | 1 | Niraparib | HER2- breast cancer, cholangiocarcinoma, gastric adenocarcinoma, pancreatic cancer with deleterious HRR mutations | Niraparib, anlotinib | not yet recruiting |  |
| NCT04753879 | 2 | Olaparib | stable or progressive PDAC post six cycles of GAX-CI | Nab-paclitaxel, gemcitabine, cisplatin, irinotecan, capecitabine (GAX-CI), pembrolizumab, olaparib | not yet recruiting |  |
| NCT04584008 | N/A | ? | metastatic gastrointestinal tumors | Arm A: matched targeted agent  Arm B: unmatched therapy | recruiting | goal of the study is to see if targeted therapies improve response |
| NCT04503265 | 1/2 | AMXI-5001 | advanced or metastatic tumors | AMXI-500 | recruiting |  |
| NCT04644068 | 1/2a | AZD5305 | advanced solid tumors | Arm A: AZD5305  Arm B: AZD5305, paclitaxel  Arm C: AZD5305, carboplatin, +/-paclitaxel | recruiting |  |
| NCT04425876 | 1 | Fluzoparib | resectable or borderline resectable PDAC | Fluzoparib, mFOLFIRINOX 🡪 maintenance fluzoparib | recruiting |  |
| NCT04228601 | 1/2 | Fluzoparib | local advanced/metastatic PDAC (g*BRCA1/2, gPALB2* mut) | Arm A: fluzoparib, mFOLFIRINOX 🡪 maintenance fluzoparib  Arm B: placebo, mFOLFIRINOX 🡪 maintenance placebo | recruiting |  |
| NCT04300114 | 3 | Fluzoparib | metastatic PDAC that has not progressed on first-line platinum-based therapy (*gBRCA1/2, gPALB2* mut) | Arm A: maintenance fluzoparib  Arm B: placebo | recruiting |  |
| NCT03404960 | 1/2 | Niraparib | locally advanced or metastatic PDAC | Arm A: niraparib, nivolumab  Arm B: niraparib, ipilimumab | recruiting |  |
| NCT03553004 | 2 | Niraparib | metastatic pancreatic cancer with germline or somatic mutations in DDR | Niraparib | recruiting |  |
| NCT03601923 | 2 | Niraparib | unresectable or metastatic pancreatic cancer (with *BRCA1, BRCA2, PALB2, CHEK2* or *ATM* mut – either germline or somatic) | Niraparib | recruiting |  |
| NCT04409002 | 2 | Niraparib | metastatic PDAC | Niraparib, dostarlimab, radiation | recruiting |  |
| NCT04493060 | 2 | Niraparib | metastatic PDAC with *BRCA1/2, PALB2, BARD1, RAD51c,* or *RAD51d* mut | Niraparib, dostarlimab | recruiting |  |
| NCT04182516 | 1 | NMS-03305293 | locally advanced/metastatic HER2- breast cancer, epithelial ovarian cancer, castration-resistant prostate cancer, pancreatic cancer (*BRCA1/2* not required for dose escalation phase, but enrichment will later be attempted in dose expansion phase) | NMS-03305293 | recruiting |  |
| NCT03878524 | 1 | Olaparib | 56 hematological and solid malignancies | selection of 2 targeted drugs | recruiting |  |
| NCT04005690 | 1 | Olaparib | PDAC | Arm A: cobimetinib (MEK/ERKi)  Arm B: olaparib | recruiting |  |
| NCT02498613 | 2 | Olaparib | NSCLC, SCLC, PDAC, TNBC | Cediranib maleate, olaparib | recruiting |  |
| NCT04548752 | 2 | Olaparib | metastatic PDAC with g*BRCA*-mut | Arm A: olaparib, pembrolizumab  Arm B: olaparib | recruiting |  |
| NCT03337087 | 1/2 | Rucaparib | Metastatic pancreatic, colorectal, gastro-esophageal and bilary cancer | Liposomal irinotecan, leucovorin, fluorouracil, rucaparib | recruiting |  |
| NCT04171700 | 2 | Rucaparib | Unresectable, locally advanced or metastatic solid tumor with deleterious mutation in *BRCA1, BRCA2, PALB2, RAD51C, RAD51D, BARD1, BRIP1, FANCA, NBN, RAD51* or *RAD51B* | Rucaparib | recruiting |  |
| NCT04550494 | 2 | Talazoparib | advanced tumors with DDR mutations | Talazoparib | recruiting |  |
| NCT03637491 | 1/2 | Talazoparib | metastatic solid tumors | Arm A: avelumab (PD-L1i), binimetinib (MEKi)  Arm B: avelumab, binimetinib, talazoparib  Arm C: binimetinib, talazoparib | terminated | Available clinical data has shown limited anti-tumor activity and reaching target study drug dose levels may not be feasible |
| NCT01233505 | 1 | Veliparib | advanced solid tumors with *BRCA1/2* mutations | Veliparib, capecitabine, oxaliplatin | terminated |  |
| NCT01282333 | 1 | Veliparib | metastatic or unresectable advanced biliary/pancreatic cancer, urothelial cancer, or non-small cell lung cancer | Veliparib, gemcitabine hydrochloride, cisplatin | terminated |  |
| NCT02511223 | 2 | Olaparib | metastatic PDAC (no g*BRCA*, but somatic mut are allowed) | Olaparib | unknown |  |
| **WEE1** | | | | | | |
| NCT02194829 | 1/2 | Adavosertib | metastatic or unresectable locally advanced PDAC | Arm A/B (phase 1): nab-paclitaxel, gemcitabine HCl, adavosertib  Arm C (phase 2): nab-paclitaxel, gemcitabine HCl, placebo  Arm D (phase 2): nab-paclitaxel, gemcitabine HCl, adavosertib | active, not recruiting |  |
| NCT04768868 | 1 | IMP7068 | advanced solid tumor | IMP7068 | recruiting |  |
| **Other** | | | | | | |
| NCT03682289 | 2 | Olaparib / AZD6738 | locally advanced or metastatic solid tumor | Arm A: AZD6738 (ATRi)  Arm B: AZD6738, olaparib | recruiting |  |
| NCT00839332 (Laquente et al., 2017) | 1/2 | Rabusertib | phase 1: metastatic cancers, phase 2: PDAC | Arm A: rabusertib, gemcitabine  Arm B: gemcitabine | completed | OS rabusertib + gemcitabine: 7.8 (5.0 to 11.1)  gemcitabine: 8.3 (5.1 to 14.1) |
| NCT03997968 | 1/2 | CYT-0851 (RAD51 inhibitor) | B-cell malignancies, metastatic breast cancer, recurrent head and neck cancer, ovarian cancer, soft tissue sarcoma, recurrent metastatic or locally advanced pancreatic cancer, advanced SCLC | CYT-0851 | recruiting | 10 patients response evaluable by DCO. 1 PaCa patient stable disease for 111 days. |
